# Patient positioning and immobilization procedures for hybrid MR-Linac systems

**DOI:** 10.1186/s13014-021-01910-6

**Published:** 2021-09-20

**Authors:** Francesco Cuccia, Filippo Alongi, Claus Belka, Luca Boldrini, Juliane Hörner-Rieber, Helen McNair, Michele Rigo, Maartje Schoenmakers, Maximilian Niyazi, Judith Slagter, Claudio Votta, Stefanie Corradini

**Affiliations:** 1 Advanced Radiation Oncology Department, IRCCS Sacro Cuore Don Calabria Hospital, Negrar Di Valpolicella, VR Italy; 2 University of Brescia, Brescia, Italy; 3 Department of Radiation Oncology, University Hospital, LMU Munich, Munich, Germany; 4 Radiology, Radiation Oncology and Hematology Department, Fondazione Policlinico Universitario “Agostino Gemelli” IRCCS, Roma, Italy; 5 Department of Radiation Oncology, University Hospital of Heidelberg, National Center for Radiation Oncology (NCRO), Heidelberg Institute for Radiation Oncology (HIRO), Heidelberg, Germany; 6 The Royal Marsden NHS Foundation Trust, and Institute of Cancer Research Sutton, Surrey, UK; 7 Department of Radiation Oncology, University Medical Center Utrecht, Heidelberglaan 100, 3584 CX Utrecht, The Netherlands; 8 Department of Radiation Oncology - Cancer Center Amsterdam, Amsterdam University Medical Centers, University of Amsterdam, Amsterdam, The Netherlands

## Abstract

Hybrid magnetic resonance (MR)-guided linear accelerators represent a new horizon in the field of radiation oncology. By harnessing the favorable combination of on-board MR-imaging with the possibility to daily recalculate the treatment plan based on real-time anatomy, the accuracy in target and organs-at-risk identification is expected to be improved, with the aim to provide the best tailored treatment. To date, two main MR-linac hybrid machines are available, Elekta Unity and Viewray MRIdian. Of note, compared to conventional linacs, these devices raise practical issues due to the positioning phase for the need to include the coil in the immobilization procedure and in order to perform the best reproducible positioning, also in light of the potentially longer treatment time. Given the relative novelty of this technology, there are few literature data regarding the procedures and the workflows for patient positioning and immobilization for MR-guided daily adaptive radiotherapy. In the present narrative review, we resume the currently available literature and provide an overview of the positioning and setup procedures for all the anatomical districts for hybrid MR-linac systems.

**Supplementary Information:**

The online version contains supplementary material available at 10.1186/s13014-021-01910-6.

## Introduction

The recent introduction of hybrid magnetic resonance (MR)-guided linear accelerators (linac) represents a remarkable innovation for the field of radiation oncology. This technology combines the advantages of enhanced MR-based soft tissue visualization with the ability to adapt the treatment plan on a daily basis, with the goal of providing the best possible treatment for the patient. Compared to conventional CT-based image-guidance, the refined imaging of MRI with optimal soft tissue contrast allows clinicians to better identify target volumes and critical structures with potentially less exposure of organs at risk [1].

Furthermore, the image-guidance is performed without any additional radiation dose exposure. This type of advanced on-board imaging is a necessary prerequisite for daily adaptive radiotherapy, where the treatment plan is re-calculated on the basis of the patient´s anatomy of the day [2].

To date, two main systems are available for clinical use: the Elekta Unity system (Elekta AB, Stockholm, Sweden) and the Viewray MRIdian system (Viewray Inc., Cleveland, USA) [3, 4].

The Unity system is based on the integration of a 1.5 T magnetic resonance scanner with a 7 MV linear accelerator and allows a daily adaptive radiotherapy applicable through two different workflows: the adapt-to-shape procedure, which requires a daily re-contouring of the target and organs at risk (OARs) prior to the generation of the treatment plan according to anatomy of the day; and the adapt-to-position strategy, based on daily update of the isocenter position, where no re-contouring is performed.

The MRIdian system combines a 0.35 T split magnetic resonance scanner with a circular ring-gantry that is positioned between the two magnets. Therefore, all 6 MV linac components are shielded to avoid magnetic field interferences [4]. The system allows to shift the couch and to predict the dose on the anatomy of the day. Moreover, both simple re-optimization or full online-adaptive workflow with dose re-optimization are available [5, 6].

However, these advanced online re-planning solutions are still burdened by longer treatment times, which may affect intra-fractional patient and especially organ motion due to the fraction duration. Patient positioning is indeed significantly different from conventional linac treatments due to the small gantry size and the need to include MRI-coils in the immobilization process. In this scenario, the need for a reliable and comfortable patient positioning is a critical feature to perform a safe and effective MR-guided treatment [7, 8].

To date, MR-guided radiation therapy (MRgRT) is in its early days, with many innovations ahead that need to be fully explored and exploited in their potential applications. Daily online-adaptive radiotherapy poses new challenges to consider and new clinical workflows need to be implemented in clinical practice, in close collaboration with other professionals involved in treatment adaptation (i.e. medical physicists and RTTs).

Given the relatively recent commercial availability of the MRI-guided systems, details on patient positioning and immobilization devices are still lacking. The purpose of this narrative review is to outline the currently available literature regarding patient setup in online MR-guided radiation therapy (oMRgRT). In addition, the authors have included their own initial clinical experience from centers equipped with the aforementioned two different systems. In order to provide the reader with a practical reference tool, all positioning devices used at the different institutions are illustrated with pictures at the end of all chapters and reported for the different anatomic regions. In addition, the Additional file 1 file provides tables of the equipment used.

### Specifications of the available hybrid MR-linac systems

The Unity treatment table has similar couch index points as used in most other conventional linacs and is therefore theoretically compatible with commercially available positioning devices. However, the treatment table itself cannot be moved for patient repositioning. A table top overlay is available for CT simulation for electron density acquisition to enable reproducible positioning of the integrated RF coil and patient set-up using the same couch index points. The maximum field size in the isocenter plane is 57.4 × 22.0 cm, while the distance from the source is 143.5 cm and the inner diameter of the gantry is 70 cm [3].

Conversely, the treatment table can move in all three dimensions in the MRIdian system, within a range of 20 cm craniocaudally from the isocenter, while lateral shifts of up to ± 7 cm are possible for patient repositioning. During treatment planning, the achievable couch positions can be displayed and taken into account for isocenter placement. The gantry is also 70 cm wide and the maximum field size is 27.4 × 24.1 cm. However, the couch is 2 m long, which might turn out to be a limited length for particularly tall patients and presents indexing notches with a distance of 20 cm [4].

**Fig. 1:**
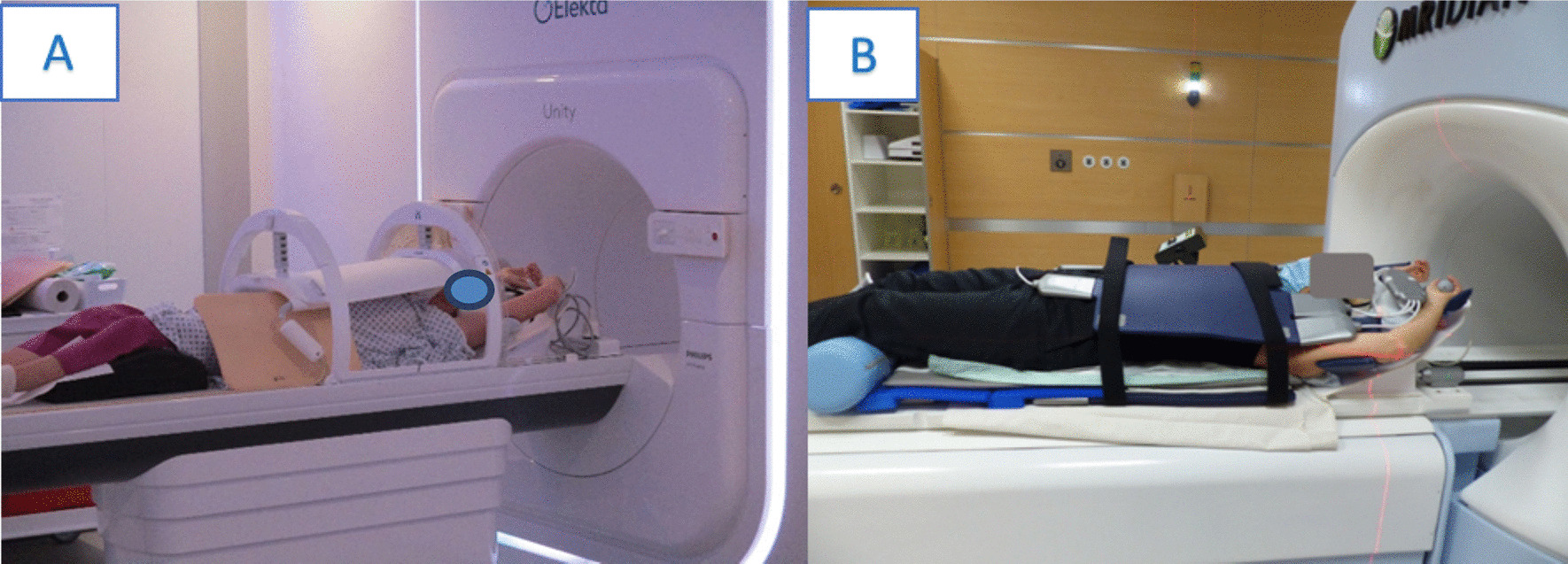
Example of MR-coils of the two commercially available MR-linac systems **a** the Unity system (Elekta AB, Stockholm, Sweden) and **b** the Viewray MRIdian system (Viewray Inc., Cleveland, USA)

Fundamental differences between the two systems are present regarding the coils. While the Unity system has coils integrated in the table, the MRIdian system uses a whole-body RF transmit coil and surface receive coils, anteriorly and posteriorly to the patient. The receive coils consist of radiolucent phased arrays with 2 × 5 channels (anterior and posterior) for head and neck and 2 × 6 channels for the torso, embedded in low-density foam and characterized by uniform attenuating characteristics (see Fig. 1). Besides the advantages in terms of versatility, servicing and easy substitution in case of need, this on-table coils setting can make the patient positioning challenging, as the posterior receive coil has always to be considered when using positioning devices and may require cushions for padding of coils electronic feedboard boxes [3, 4].

Moreover, as reported by Barnes et al., the absence of lateral lasers in the Unity system and the close proximity to the coils in the MRIdian system mean that minute adjustments to patient positioning to align to lasers are not possible. Rather patient positioning is focused on general patient comfort and positioning and may be as effective [9].

With respect to positioning devices, MRI safety aspects are obviously of paramount importance. In addition to adequate patient screening for MR-compatibility, all equipment must be designed and tested for a dedicated use in a MR environment. The equipment must be approved for ferromagnetic safety, which is usually already confirmed by the manufacturer. However, it is recommended that the safety status of each device is verified as part of an on-site QA process to test ferromagnetic properties, imaging artefacts, dose attenuation level and physical compatibility with the coils.

All MR conditional equipment should be labeled to avoid mix-ups with non-MR compatible positioning devices. However, most manufactures already use designated colors to avoid this sort of complication. Other issues to be considered for patient positioning are the limited gantry size (70 cm), which imposes additional restrictions compared to conventional radiotherapy patient positioning. In particular, for obese patients or when patients are positioned with both arms above the head, the remaining space to the bore wall might be very small. In some cases, this configuration may not be possible at all and patients need to be positioned with their arms parallel to the body. An additional consideration is the approach taken when there is machine breakdown. If the patient is to be treated on a conventional linac, the immobilization systems must be transferrable and staff familiar with them.

Another point to consider is that the MR-specific equipment must be integrated into patient positioning. In addition to the already mentioned coils, patients need hearing protection (earmuffs and/or earplugs) and an emergency squeeze bulb or push-button alarm. Moreover, the patients need to be instructed on how staff will communicate with them during treatment delivery. Overall, because of the longer treatment times in adaptive MRgRT, the patient set-up should be as comfortable as possible to increase compliance and reduce intrafraction patient movement and potential claustrophobic reactions. For this reason, in some centers the use of prism glasses and a TV screen outside the bore are applied to make more pleasant the stay in the treating room. Of note, this device cannot be applied for head and neck treatments. (see Additional file 1).

A thorough clinical evaluation to assess the expected compliance is therefore strongly encouraged, especially in case of frail and elderly patients which may present borderline general conditions [10].

Particularly in this scenario, the relatively longer treatment time raises the issue whether preferring a rigid immobilization in order to reduce as possible intra-fraction motion or a non-rigid immobilization to increase patient’s comfort and compliance to the treatment, also in light of the necessary presence of the coil in the immobilization phase. Of course, although recent novel technological devices have reduced the need for rigid immobilization, in some anatomical districts, the use of rigid immobilization systems still remains irreplaceable. Since MRgRT is in its infancy, we believe that future data will provide stronger evidence in favor of the optimal positioning strategy, as it is presumable that the refined accuracy in image guidance will lead to a lesser use of rigid immobilization tools.

However early reports indicate that patients tolerate MRI treatments very well and the most common reported issues have been due to the cold environment and noise [11, 12].

## Clinical sites

### Brain and head-and-neck

The application of MRgRT for brain tumors represents a potential opportunity to exploit the advantages provided by the use of MRI-imaging, not only for target volume delineation but also for its functional assessment. In addition, MR-guided adaptive treatments may be useful to adjust target volumes during the course of treatment, for example in head-and-neck tumors in the case of tumor shrinkage during chemoradiation or resection cavities in brain tumors [13–16].

For radiotherapy treatments of the brain and head-and-neck region, the use of thermoplastic masks remains the gold standard to prevent motion of the head and guarantee reproducible patient positioning [17].

In the particular environment of MRgRT, it is principally challenging to perform patient immobilization that includes proper coil positioning and hearing protection in addition to the thermoplastic mask. To date, many institutions have created their own in-house developments to allow proper coil placement. However, there are already some dedicated systems commercially available (see Additional file 1: Table 1).

To date, there is limited evidence available from preliminary reports of MRgRT treatments for brain malignancies [18]. While there are some experiences using radiotherapy positioning devices in diagnostic MRI scanners to obtain diagnostic imaging in treatment position [19], evidence from hybrid MR-linac systems is scarce. Moreover, none of the available hybrid systems provides specific brain coils for dedicated imaging.

One of the important factors that must be taken into account for brain and head-and-neck MRg RT, is the potentially longer treatment delivery time in the framework of oMRgRT. Especially in the case of head-and-neck irradiation in patients with a tracheostoma, breathing or mucus-related coughing might be problematic. This could potentially also influence MR image quality due to the presence of artefacts. Patients comfort must be therefore be preserved in order to keep inter- and intra-fraction motion as low as possible. Moreover, the use of fast imaging sequences might be advantageous in this setting [20].

To date, only the study by Chen et al. [21] reported details of patient immobilization for the head-and-neck region during MRgRT. In a cohort of 18 patients diagnosed with head and neck tumors, immobilization was performed using a thermoplastic mask system with a custom modified Timo cushion (S-type, Med-Tec, Orange City, IA, USA) that fitted the MR receive coil. The mask was then fixed on an indexed plastic board through the cut outs of the coils.

**Fig. 2:**
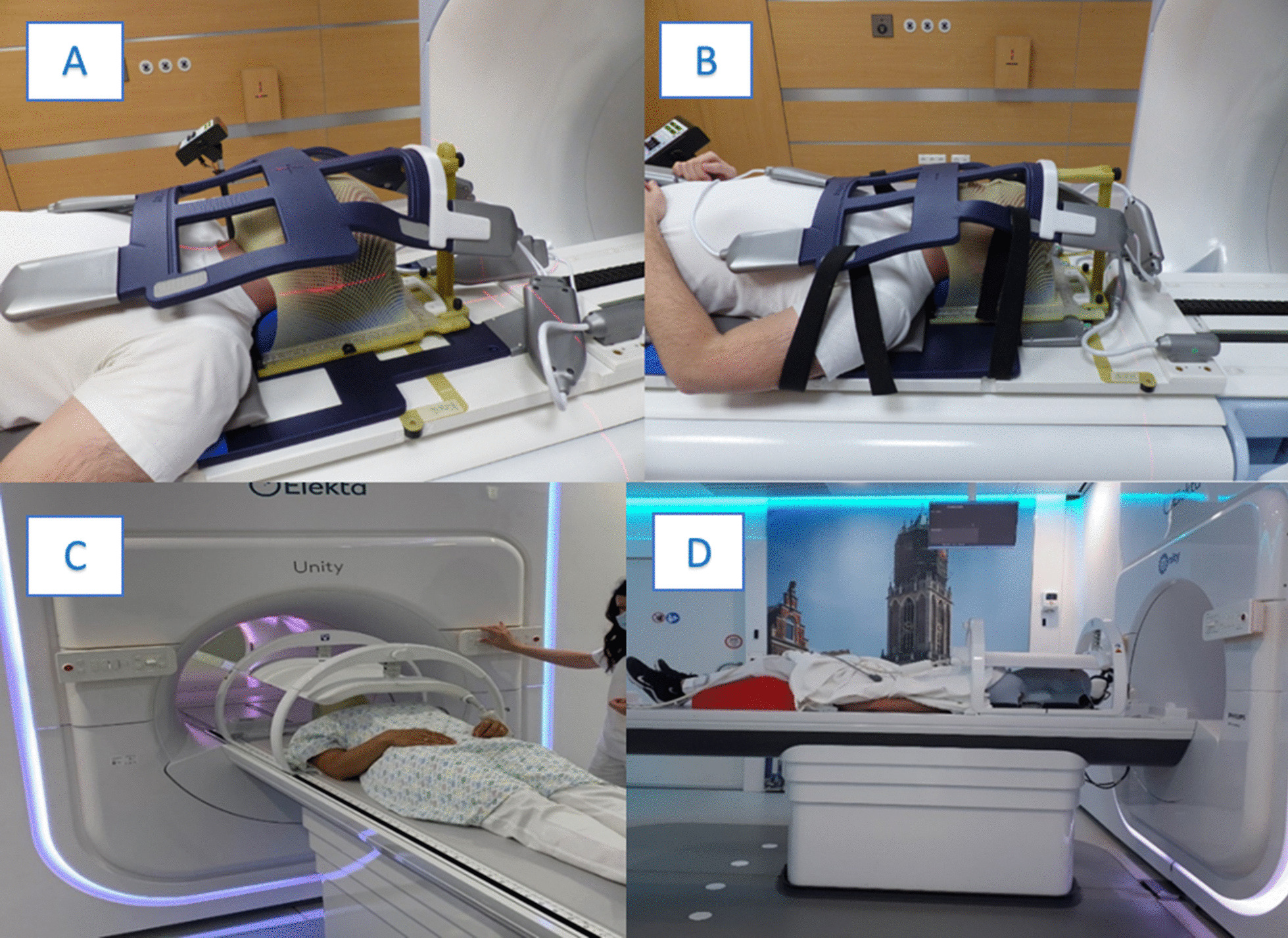
Examples of patient positioning for **a** brain and **b** head&neck radiotherapy using the MRIdian system (Viewray Inc., Cleveland, USA) and **c**, **d** using the Elekta system (Elekta AB, Stockholm, Sweden)

Examples of the systems used at the contributing institutions can be found in Fig. 2. Figure 2 (A) shows an example of the immobilization used for brain irradiation with the MRIdian system. In this case the so-called head and neck coils are used. The posterior surface receive coils (flat without plastic bar) are positioned on the table and the HeadSTEP UP VR system (IT-V, Innsbruck, Austria) is placed on top of the coil and fixed on the table using appropriate indexing bars. The patient`s head is positioned with appropriate MR-compatible pillows and fixated with a custom made thermoplastic mask (IT-V, Innsbruck, Austria). Then the anterior receive coil is positioned at the top and hooked into the HS Flexcoil holder VR of the HeadSTEP system in order to avoid touching of the patient`s face. The setup for head and neck MRgRT is similar and an example is shown in Fig. 2 (B). Since the field of view for MR imaging must be further inferior in head and neck irradiation than for cerebral RT, the torso coil is used as posterior coil and a dedicated HeadSTEP UP VR H&N system is mounted above it, with a longer flexi-coil holder for adequate positioning of the anterior receive H&N coil.

Figure 2 (C) and (D) shows examples of patient positioning using the Unity system. Patient immobilization is performed in supine position with the arms along the body. The customized thermoplastic mask (IT-V, Innsbruck, Austria) is mounted to the indexed HeadSTEP MR system (Elekta, Stockholm, Sweden), with the head of the patient positioned on a MR-compatible pillow. Then the coil is positioned and fixed to the table.

## Thorax

### Lung

The thorax is a challenging site for MR-guided radiotherapy because of an increased risk of image artefacts due to organ motion, unless imaging is performed in breath-hold conditions [22, 23].

MRI is however an attractive tool for better visualization of critical structures and organs at risk, such as the brachial plexus or the heart substructures, and offers improved accuracy of the target position by online intrafractional tumor visualization.

To date, the use of MR-guided stereotactic body radiotherapy (SBRT) has been recommended for selected cases, like central or ultra-central tumors, where a dosimetric advantage has been reported compared with conventional linac plans, or in the setting of re-irradiation [24, 25]. Another attractive indication is single-fraction SBRT, in which healthy lung tissue can be spared by using a respiratory gated breath-hold technique with smaller margins than by using a classical ITV approach [26].

In all the available experiences, a crucial feature is the minimization of the respiratory-induced motion, which is usually controlled by active approaches like gating, tracking or active breathing control [27].

Furthermore, MR-linacs are equipped with cine MR imaging, obtaining up to 8 frames per second on the MRIdian units, which allows online visualization of the tumor motion with the aim to reduce uncertainties about the target trajectory during the respiratory phases or to perform anatomy tracking. This feature is intended to replicate the role of a 4D CT traditionally used for conventional linacs, although active gating approaches are strongly advocated for all lesions affected by respiratory motion [28].

**Fig. 3:**
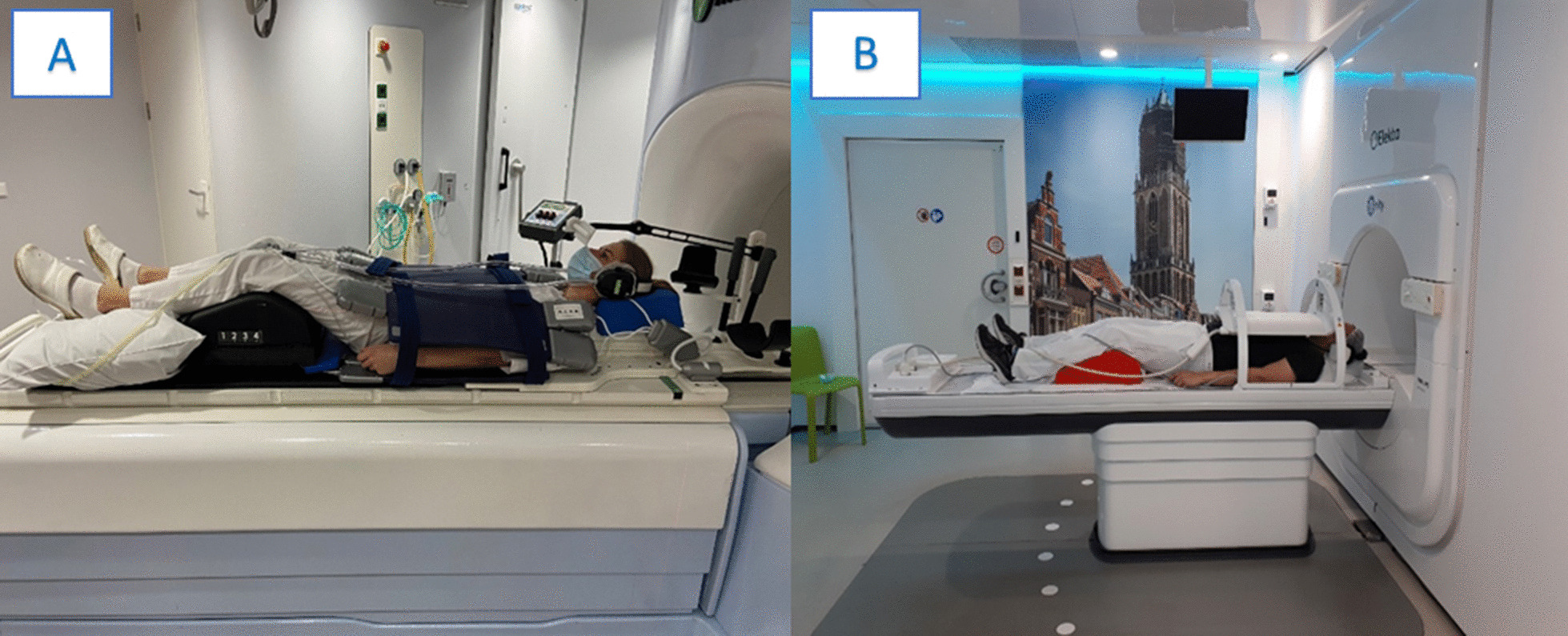
Examples of patient positioning for thorax radiotherapy using **a** the MRIdian (Viewray Inc., Cleveland, USA) and **b** Elekta system (Elekta AB, Stockholm, Sweden)

Most of the currently available reports discuss early clinical experiences and do not mention or specify any immobilization devices [29–32]. Henke et al. [33] report the results of a phase 1 trial, in which 5 patients affected by ultracentral thorax malignancies were treated. The authors describe the use of a customized immobilization per standard clinical protocol, without further details and the application of exhale breath-hold approach for respiratory gating purposes. A recent paper by Sayan et al. [34] evaluating patient-reported outcomes measures (PROMs) in a cohort of 90 patient treated with MRgRT, including 18 thoracic cancers patients, described the use of prism glasses for gating activities when a respiratory motion management was performed. As reported by other early clinical experiences, these real-time visual feedback systems facilitate voluntary breath-hold delivery during the correct respiratory phase and do not affect patient compliance [35, 36]. Examples of the contributing institutions can be found in Fig. 3.

### Breast

Concerning breast cancer, MRgRT is applied in the adjuvant setting for partial breast irradiation, or also in the neoadjuvant setting within dedicated research protocols, thanks to the better visualization of the tumor allowed by MR guidance [37, 38].

A recent position paper by Koerkamp et al. [39] has outlined the main problems of patient positioning for MR-guided breast radiotherapy. In particular, both supine and prone positions present practical challenges as the coils must be included in the positioning process without compromising the whole body contour, which is necessary for treatment planning. In addition, organ motion must also be considered; although Ahn et al. [40] reported that prone position is the optimal choice for minimizing thoracic respiratory motion and consequently artefacts generation; the study by Batulamai et al. observed no relevant impact of patient position on image quality and motion artefacts, whether in prone or supine position [41].

Preliminary experiences with MRgRT for breast cancer are available and report substantial reproducibility of the treatment [42, 43]. Further data in terms of clinical outcomes and toxicity rates are awaited from ongoing clinical trials.

Fischer-Valuck et al. [44] described their experience in the treatment of breast cancer, accounting for 26% of the cases treated with the MRIdian system in the first 2.5 years of activity. Unfortunately they did not provide any detailed information regarding patient positioning in the article. Nachbar et al. [45] reported the first case of partial breast irradiation (PBI) treated with a 1.5 T MR-linac. Both planning CT and MRI were performed in supine position with the use of a positioning device in free breathing. The article also highlights the electron air stream effect (ESE), which can lead to out-of-field dose deposition and the electron return effect (ERE), which may result in increased dose to the skin and at air/tissue interface. Especially in breast RT, where the target volume directly involves the skin, these effects can cause an increase dose to the skin and also out-of-field skin dose on the chin. Thorough plan optimization and bolus placement on the chin are emphasized. This effect is less pronounced in 0.35 T systems [46].

Charavghandi et al. [47] conducted a dosimetric study to determine the best treatment position for performing neoadjuvant PBI. They reported more favorable dosimetric endpoints of OARs when simulations were performed in prone position. More specifically, immobilization was performed with the CDR® prone breast board, while 2 patients in supine position were simulated using the Thorawedge board® and the remaining 8 patients using the Macromedics® breast board. Standard prone breast MR coils are unsuitable for hybrid MR-linac systems; for this reason, dedicated coils for MR-linacs are used for radiation delivery [48, 49].

**Fig. 4:**
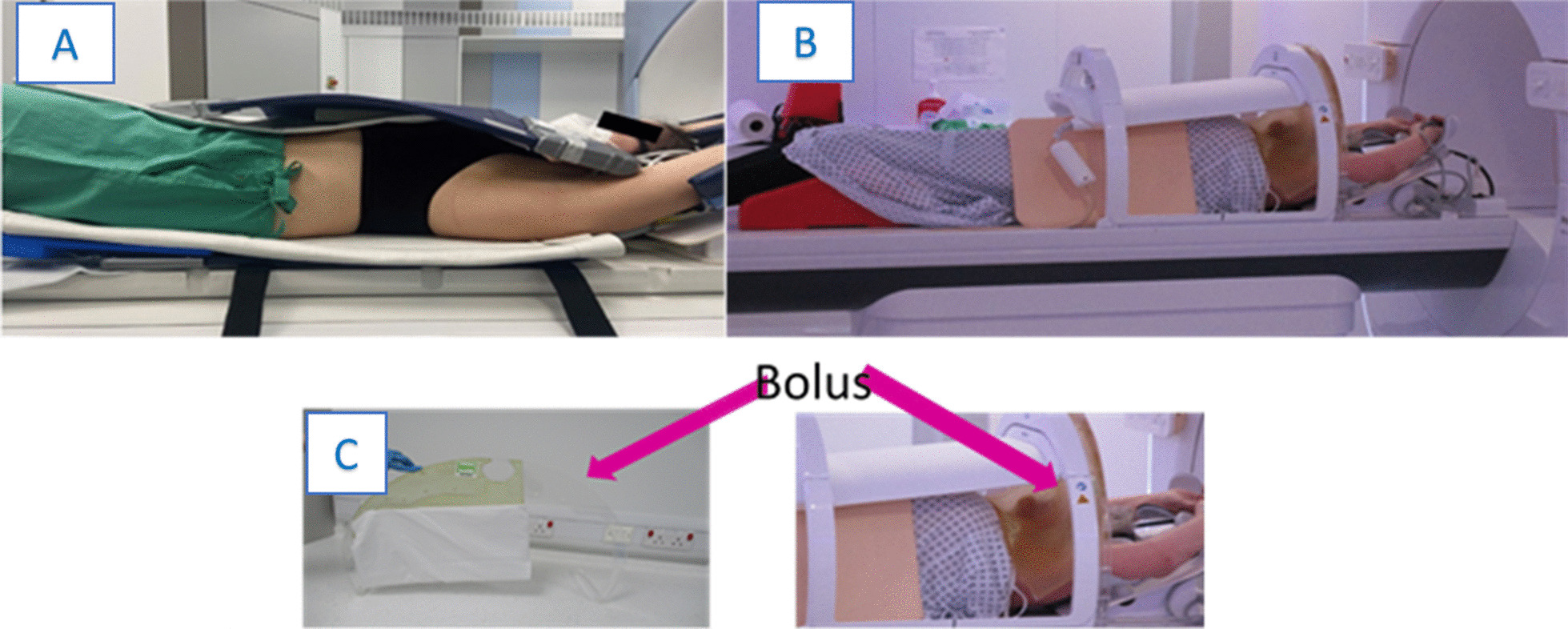
Examples of patient positioning for breast radiotherapy using the **a** MRIdian (Viewray Inc., Cleveland, USA) and **b** Elekta system (Elekta AB, Stockholm, Sweden) with **c** Bolus placement

Figure 4 (A) illustrates an example of patient positioning using the MRIdian Linac from Viewray. Patient immobilization is performed in supine position with the help of a wingstep system. The receiver surface coils are placed below the body and above the patient’s chest. A sytrofoam block is put in the intermammary cleft to keep the coils above the patient’s surface for avoiding physical deformation of the breast tissue. Coils are only attached to each other on the non-treated side to further prevent tissue deformation. Patients are also asked to wear a bra without additional support wire to ensure reproducible breast position during each treatment day.

On the Unity MR-linac (Fig. 4B) patients are positioned supine, on the Elekta wing board (Elekta AB, Stockholm) with arms supported on the arms rests. The anterior coil is positioned as close to the patient as possible and usually restricted because of proximity of the chin/nose. There can be an increased risk of out of field skin doses due to the Electron Streaming Effect (ESE). Bolus can either be laid directly on the patients skin surface or one centre created a practical, non patient-specific, shielding solution using a frame to support a bolus curtain between the treatment area and areas at risk from high ESE doses (Fig. 4C) [50].

## Abdomen

Liver lesions represent another attractive target in MR-guided radiotherapy and pose similar challenges to thoracic lesions, in terms of organ motion assessment, image distortion uncertainties and positional errors during imaging in free breathing. Moreover, in the upper abdomen, similar to the thoracic region, the occurrence of air/soft tissues interfaces is a common finding, emphasizing the need for an absolutely reliable setup procedure [51].

A detailed description of the simulation workflow for liver MR-guided SBRT was recently reported by Witt et al. [52]. However, the authors did not mention specific immobilization devices in detail. The receive coils were placed in an anterior to posterior orientation around the patient, and a contrast-enhanced MRI was acquired for optimal visualization of the target, including sagittal cine MRI images for online target lesion tracking. Preliminary reports describe encouraging results in terms of clinical outcomes and patient tolerability when respiratory gating is available, as in the case of the MRIdian system [53–58].

Similar as to the thoracic region, the availability of a 4D-MRI is a really attractive solution when anatomy tracking is not available for gated SBRT delivery. To date, the Unity system supports only non-gated treatments delivered in free-breathing, as described by Hall et al. [54]. Recently, a 4D-MRI driven workflow was evaluated for motion management in free-breathing abdominal SBRT. In a study by Paulson et al. [53] this workflow was successfully employed in a small cohort of 11 patients. The authors used an ITV approach based on 4D-MR images, while Gani et al. [59] used information from 4D-CT imaging and additional expiration breath hold and free-breathing MRI scans in a series of 10 patients with liver oligometastases. Compared to gating or tracking strategies, the ITV concept allows a faster treatment delivery, but at the cost of a larger irradiated volume [60]. Nonetheless, both techniques allow the delivery of ablative doses while sparing adjacent OARs, for example also in the treatment of pancreatic cancer [61].

Adaptive workflows are typically used to account for interfractional variation in anatomy. However, re-contouring and dose optimization extend the duration of each treatment session. As described by Boldrini et al. [62] specifically in the case of pancreatic cancer, this can be a limiting factor in the adaptive workflow process due to the proximity and constant variation of healthy structures such as intestinal loops. Consistent with this, a preliminary clinical experience by Tyran et al. [63] focused on the need for daily online plan prediction to assess dose exposure and compliance with constraints of organs at risk. They found that visual inspection of OARs was not reliable for pancreatic SBRT and that contour deformation and re-contouring was necessary to reliably predict the dose exposure and safely perform ablative treatments. Regarding treatment simulation and patient immobilization, in the study by El-Bared et al. [64], 10 patients treated with the MRIdian system for pancreatic cancer were positioned supine with the arms above the head in a MRI wing-board equipped with a surface coil array. Treatment delivery was performed during inspiration breath-hold phase with real-time MRI-based tumor tracking and automated gating.

SBRT performed with an MRI-Linac is also an attractive treatment option for adrenal gland metastases or renal cell cancer [65, 66]. Available data report the use of online-adapted respiratory-gated MRgRT without the need for special immobilization devices. All patients were advised to fast for at least 2 h before simulation and each treatment session. Nevertheless, very large changes in gastric position were reported in the case of left-sided adrenal gland treatments, suggesting a role for supportive dietary instructions in these particular cases [67, 68].

**Fig. 5:**
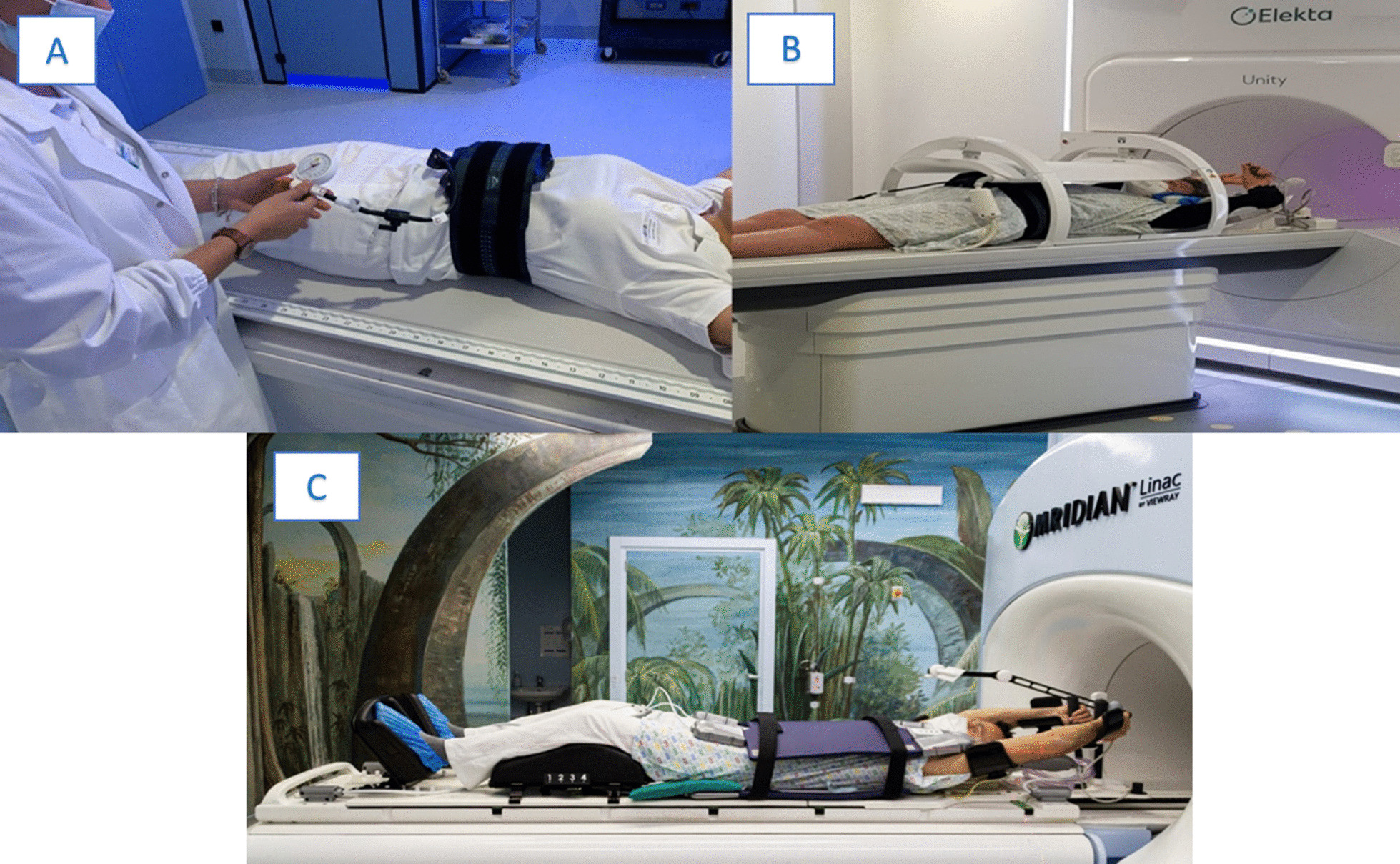
Example of patient positioning for abdomen radiotherapy using **a**, **b** the Elekta system (Elekta AB, Stockholm, Sweden) and **c** the MRIdian system (Viewray Inc., Cleveland, USA)

In Fig. 5 (A) and (B) we report an example from one of the contributing institutions concerning the treatment preparation of an adrenal gland target. Patient positioning was performed in a supine position with the arms elevated above the head. To mitigate respiratory motion, abdominal compression was performed using the pressure belt ZiFix (QFix, Avondale, USA) both, for the simulation and treatment delivery. MR-immobilization was completed with the positioning of the coil mounted on the table.

Figure 5 (C) shows an example of patient positioning for liver SBRT using the Fluxboard device (Macromedics, Moordrecht, The Netherlands). The patient was treated in supine position with both arms above the head, elbows and wrists resting on specific supports. The use of knee-support foam allows to bend the legs, increasing the comfort for the patient, while the feet resting in the appropriate support allows to reduce the rotational uncertainties. A thin foam cushion was placed on the lower end of the coil, to avoid contact between the rigid parts of the coil (i.e. feedboard box) and the patient. For patients who are not compliant with this setup, the possibility of keeping the arms along the body can be considered, avoiding beams crossing that body sector.

## Pelvis

### Prostate

Prostate is one of the more favorable anatomic sites for MRgRT, given the ability to optimally monitor daily inter- and intrafractional variations of the target and anatomic variations of adjacent healthy structures [69–73]. Moreover, the possibility to rely on MRI-imaging allows clinicians to hypothesize focal boost protocols or radiomics investigational studies [74, 75].

Preliminary experiences report an excellent feasibility of MRgRT for prostate cancer, despite a potentially longer treatment duration. Common simulation procedures with standardized protocols for bladder filling and rectal emptying have been reported in the literature. [76]

Bruynzeel et al. [77] conducted a phase II trial and performed MR-guided adaptive SBRT with 5 × 7.25 Gy and urethral sparing, describing excellent early results with low incidence of GI and GU toxicity, both in clinician- and patient-reported outcome measurements.

The prolonged RT duration does not appear to affect patient compliance or treatment tolerability, as recently highlighted in an article by Mazzola et al., who performed a PROMs evaluation in a cohort of 40 elderly patients, who may be more susceptible to suffering from time-consuming procedures [78].

However maintaining a ‘comfortably ‘ full bladder for an extended period of time may be an issue and cause an interruption to the treatment if the patient has to empty prior to treatment commencing. In addition, some authors hypothesize that the increased treatment time due to the daily adaptive planning process may compromise the dosimetric quality of the treatment, as re-optimization is performed on an anatomy of 20–40 min earlier and does not take into account the continuous displacement of the prostate and organs at risk. In this case, either an additional verification image can be acquired and a dose shift performed using the ATP workflow (Unity system), or tracking and gating can be used (MRIdian system). In cases where online tracking is not available, a margin reduction strategy should be used with caution, given the remaining uncertainties [79].

Another point that can be considered for prostate radiotherapy is the possibility of implementing rectal spacers to limit prostate motion and influence OAR dose exposure. Especially in the setting of extreme hypofractionated stereotactic radiotherapy, the use of rectal spacers has been described to achieve superior dosimetric rectal sparing [80].

Also in the case of MR-guided SBRT, the use of this devices results in a statistically significant lower dose exposure of the rectum with an expected benefit in terms of toxicity incidence. Of note, the implementation of the rectal spacer in the treatment preparation workflow had no impact on patient quality of life, as recently reported in the literature [81].

**Fig. 6:**
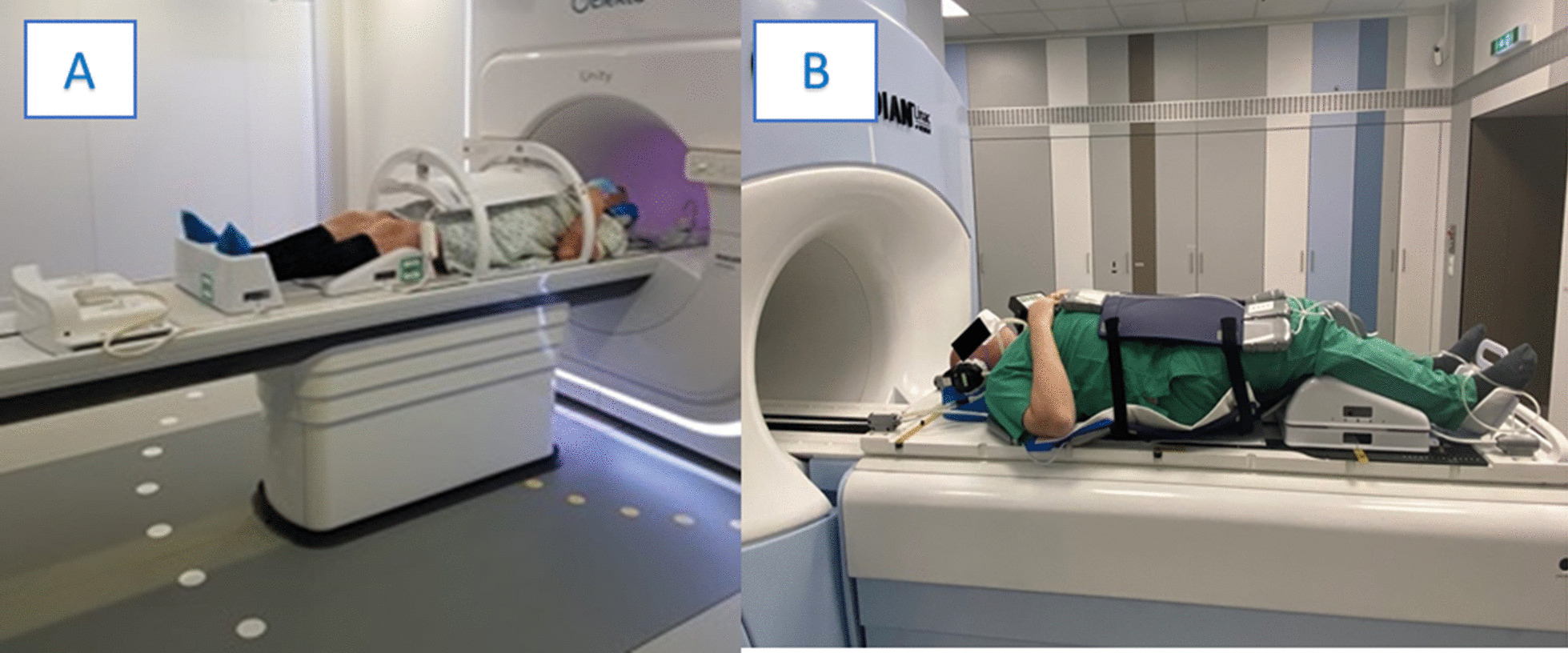
Example of patient positioning for prostate radiotherapy using **a** the Unity system (Elekta AB, Stockholm, Sweden) and **b** the MRIdian system (Viewray Inc., Cleveland, USA)

Figure 6 (A) depicts the patient positioning for the pelvic anatomic district in one of the collaborating institutions. Following the internal protocol for rectal emptying and a comfortably full bladder, the patient was immobilized in a supine position and flexed legs with the help of the KneeSTEP and FeetSTEP MR positioning device (Elekta, Stockholm, Sweden). It is also possible to use a combifix system once it is ensured any metallic screws are replaced with plastic. The coil is positioned anteriorly and fixed to the table. (see Additional file 1).

Figure 6 (B) illustrates patient positioning at the MRIdian Linac for prostate cancer treatment. The patient is immobilized in supine position using the ProsSTEP PC MR device (IT-V, Innsbruck, Austria). Receiver coils are placed below the body and on the patient’s pelvic region. Arms are positioned on the thorax. Patients are generally treated with preferably empty rectum and optimally half full bladder.

### Rectum

MR-guided radiotherapy is also a promising tool in rectal cancer, as MRI is considered the imaging gold standard for both pre- and post-treatment staging [82].

Furthermore, in the setting of locally advanced disease, the use of on board MRI to build volumetric and radiomics-based predictive models for early detection of pathological complete response is an attractive option for the treatment of this patient population, as previously suggested by different experiences [83, 84].

In addition, MRI-guided image-guidance allows clinicians to evaluate the potential adoption of intensified treatments. The first clinical report of MR-guided radiotherapy for rectal cancer describes the outcomes of 22 patients in a study by Chiloiro et al.: the patient positioning workflow consisted of a supine immobilization using the Fluxboard device (MacroMedics, Moordrecht, The Netherlands) in a fully customized configuration, using a bladder filling protocol according to institutional procedures. The authors reported a promising pathological complete response rate of 27.3%, although five patients experienced a G3 acute toxicity requiring day-hospitalization for supportive care, as a higher BED dose of 55 Gy in 25 fractions was applied in the neoadjuvant setting [85].

Nevertheless, this initial experience can be considered promising and further studies are awaited to evaluate the potential advantages provided by MR-guided radiotherapy in rectal cancer [86].

### Other

MRI is the imaging modality of choice for the diagnosis, staging and response evaluation of gynecological cancers, particularly cervical cancer [87]. The integration of MRI in the treatment planning procedures allows a better visualization of the tumor and pelvic OARs due to the optimal soft tissue contrast [88]. In brachytherapy, the MR-based adaptive target concept, which takes into account the topography of the primary tumor at diagnosis, as well as the regression observed during external beam radiotherapy, is already state of the art and leads to better tumor control, increased survival and decreased treatment toxicity [89].

Similar to other pelvic targets, treatment planning for gynecological malignancies must take into account the daily anatomical variations of adjacent healthy structures. At the same time, especially in the case of cervical cancer, MRI allows clinicians to improve the accuracy of tumor identification. To date, there is only a small series published by Boldrini et al. [90] reporting preliminary data on the use of MR-guided radiotherapy for locally advanced cervical cancer. In their study, the authors report the outcomes of nine patients enrolled in an institutional study protocol of neoadjuvant chemoradiation delivered with the MRIdian system. Patient immobilization was performed using the Fluxboard device (Fluxboard, MacroMedics, The Netherlands) as well as dedicated positioning devices. For treatment planning, simulation was performed with a reproducibly full bladder and empty rectum. Intrafractional motion management consisted of a GTV-based gating approach using real-time cine-MRI in the sagittal plane. No constraints violations were recorded in this series, with no severe acute toxicities and comparable outcome to patients treated on a conventional linac. Considering the limitations of this study, as neoadjuvant chemoradiation before surgery is not a standard of care in cervical cancer, the authors emphasize the role of MR-guided RT in detecting potential tumor shrinkage or an MR-guided SBRT boost approach in the case of patients unfit for a brachytherapy boost [91].

In the pelvic region, MR-guided radiotherapy is also considered an attractive treatment option for oligometastatic disease. Especially in the case of lymph node oligometastases, the advantages of online adaptive radiotherapy combined with a refined image guidance system allows clinicians to propose more aggressive schedules in terms of extreme hypofractionation [92, 93]. This is of particular interest, for example, in targets very close to intestinal loops, which are frequently prone to remarkable anatomical positioning changes from one fraction to another and potentially also within the same fraction. As already reported in early experiences, SBRT delivered with MR-linac systems in this subset of patients is feasible and safe [94–96].

Especially in the case of SBRT, where a small treatment volume is planned to receive very high doses with a rapid dose fall-off outside the PTV to spare the OARs, accuracy of patient positioning is a crucial factor for a safe and effective treatment. Historically, the use of vacuum cushions for SBRT has been reported as a method to improve accuracy in the daily setup [97]. Technological advances and refinements in image guidance have reduced the role of vacuum cushions for SBRT, since the customization of the cushions is a time consuming procedure and it also presents a logistic issue for storage, leading to a progressive drop in the use of this tool for SBRT treatments. However, it is still in clinical use in many centers. In a recent study by Werensteijn-Honingh et al. [98], the authors investigated the potential impact of a vacuum cushion on intrafractional lymph-node motion in a comparison of 38 patients receiving lymph node SBRT with or without a vacuum cushion-based immobilization (BlueBAG BodyFIX 14 Rectangular 700 × 1825 mm/50L, Elekta AB). During initial image acquisition, significantly smaller absolute translational deviations in the antero-posterior direction were observed for the GTV and bony anatomy in the cohort of patients treated with the vacuum-cushion. Interestingly, the use of the vacuum-cushion had no effect on intrafractional motion during the delivery phase, and might therefore be safely omitted for MR-guided SBRT of lymph-nodes oligometastases, as daily adaptive recontouring, reoptimization and position verification imaging prior to the delivery phase can adequately compensate organ motion uncertainties.

## Conclusions

Hybrid MR-linac systems represent a new concept in radiation oncology and their role is expected to be constantly growing in the coming years. Currently, there is limited clinical data available in the literature due to the novelty of this technology, and the details regarding patient immobilization and treatment setup are even more scarce. Available preliminary experiences indicate the feasibility of MRgRT in various anatomical regions with promising clinical results. However, patient positioning is significantly different from conventional linac treatments due to the small gantry size and the need to include MRI-coils in the immobilization process.

Moreover, as actively gated and online adaptive treatment sessions’ duration is usually longer than conventional RT ones, patient preparation and positioning becomes critical to ensure a safe and effective MR-guided treatment.

## Supplementary Information

**Additional file 1.** Tables and figures.

## Data Availability

Not available.

## Abbreviations

ATP: Adapt to position
ATS: Adapt to shape
BED: Biologically equivalent dose
CT: Computed tomography
ESE: Electron streaming effect
GI: Gastrointestinal
GU: Genitourinary
GTV: Gross tumor volume
ITV: Internal target volume
MRI: Magnetic resonance imaging
MRgRT: Magnetic resonance-guided radiation therapy
OAR: Organ at risk
PROMs: Patient-reported outcome measures
PTV: Planning target volume
RTT: Radiotherapy technologist
SBRT: Stereotactic body radiotherapy
