# Supplementary material for: Patient positioning and immobilization procedures for hybrid MR-Linac systems
Source: Radiat Oncol. 2021 Sep 20;16:183. doi: 10.1186/s13014-021-01910-6 (PMC8454038; doi:10.1186/s13014-021-01910-6)
Supplement: Supplementary file 1 — Additional file 1. Tables and figures. [file 13014_2021_1910_MOESM1_ESM.docx]

**Supplementary Material**

1. **Brain/ Head & Neck immobilization**

**Table 1 – List of exemplar devices used for Brain/Head & Neck immobilization**

| **Therapy region** | **Positioning devices** | **Vendor** | **MR-linac device** |
| --- | --- | --- | --- |
| Brain | HeadSTEP UP VR system | IT-V, Innsbruck, Austria | Viewray |
| Head & neck | HeadSTEP UP VR H&N system | IT-V, Innsbruck, Austria | Viewray |
| Brain / Head & neck | HS Flexcoil Holder VR | IT-V, Innsbruck, Austria | Viewray |
| Brain / Head & neck | Thermoplastic mask | IT-V, Innsbruck, Austria | Viewray |
| Brain / Head & neck | Flexicoil Comfort Modul VR cushion for flexicoil electronic boxes padding | IT-V, Innsbruck, Austria | Viewray |
| Brain / Head & neck | Fluxboard (dedicated Head & neck module) | Macromedics, Moordrecht, The Netherlands | Viewray |
| Brain / Head & neck | Emergency squeeze bulb | Viewray, USA | Viewray |
| Brain / Head & neck | Thermoplastic mask | Macromedics , Moordrecht, The Netherlands | Unity |
| Brain | Head&NeckSTEP M Unity system | Elekta, Stockholm, Sweden/ IT-V, Innsbruck, Austria | Unity |
| Head & neck | ExaFix-5A, acrylic baseplate, 5-point fixation, MR safe | MacroMedics, Moordrecht, The Netherlands | Unity |
| Head & neck | Individual Head Support, Posifix, natural position, MR Safe | CIVCO Radiotherapy, USA | Unity |
| Head & neck | Posicast, thermoplastic 5-Point Mask, Posifix | CIVCO Radiotherapy, USA | Unity |
| Head & neck | KneeSupport foam | MacroMedics, Moordrecht, The Netherlands | Unity |
| Head & neck | Earplugs E-A-R soft | 3M, USA | Unity |
| Head & neck | Emergency squeeze bulb | Elekta, Stockholm, Sweden | Unity |

**Figure 1 – Example for Brain/Head & Neck immobilization (without patient)**


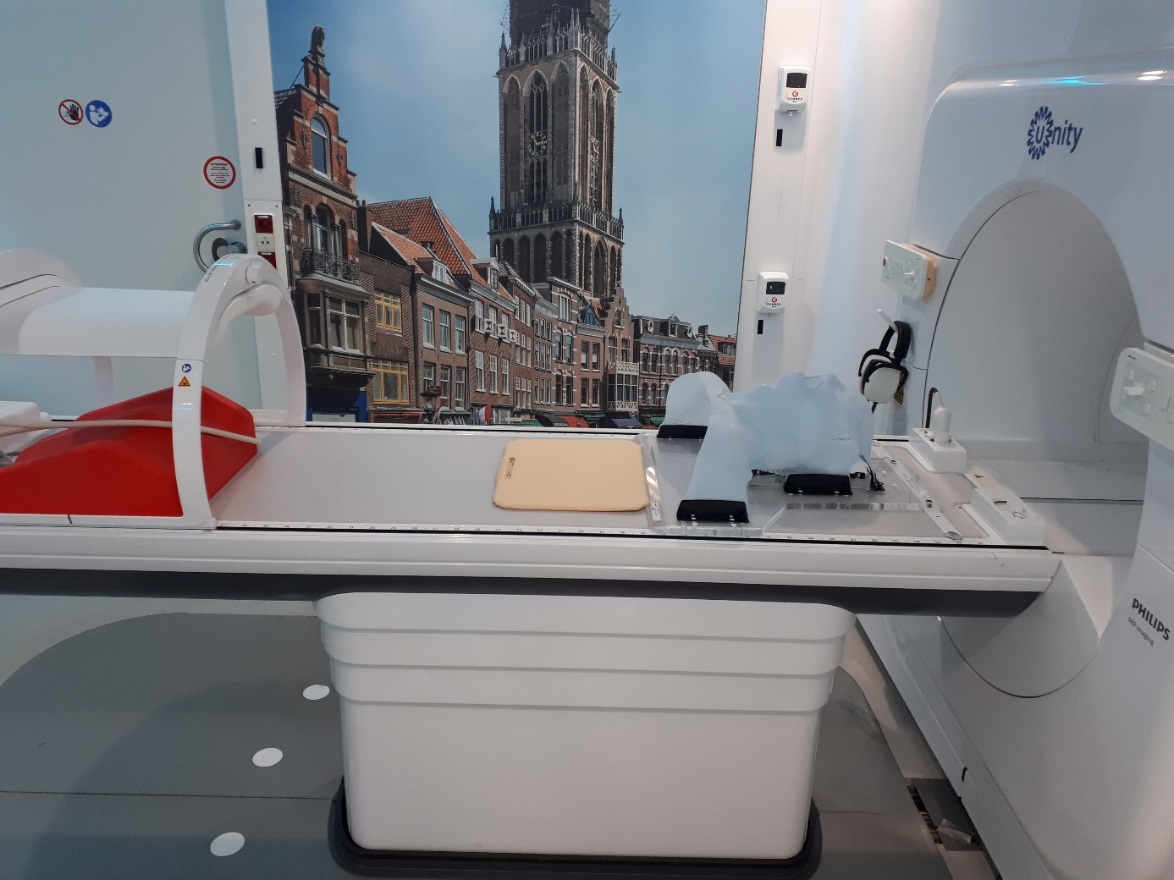


*Courtesy of UMC Utrecht – Unity Elekta MR-Linac. Used devices: ExaFix-5A acrylic baseplate attached with indexbar on table index position, individual Head Support Posifix, thermoplastic 5-Point Mask, KneeSupport™ foam (without indexbar), anterior Elekta coil bridge, Earplugs, Emergency squeeze bulb. Immobilization description: Prior to each treatment, for all the anatomical districts, patients are positioned on the treatment couch in the predefined supine index position with the help of an in-room laser system (Micro Linac & MR laserlight, Gammex). Specifically for the brain/head&neck district, we use the marks on thermoplastic mask. Two four-channel receive arrays are used for imaging. The posterior coil is positioned under the patient couch, while the anterior coil is placed on an indexed coil bridge as close to the patient as possible to maximize the signal-to-noise.*

**Figure 2 – Devices for Brain (A) and Head & Neck immobilization (B) (without patient)**

*
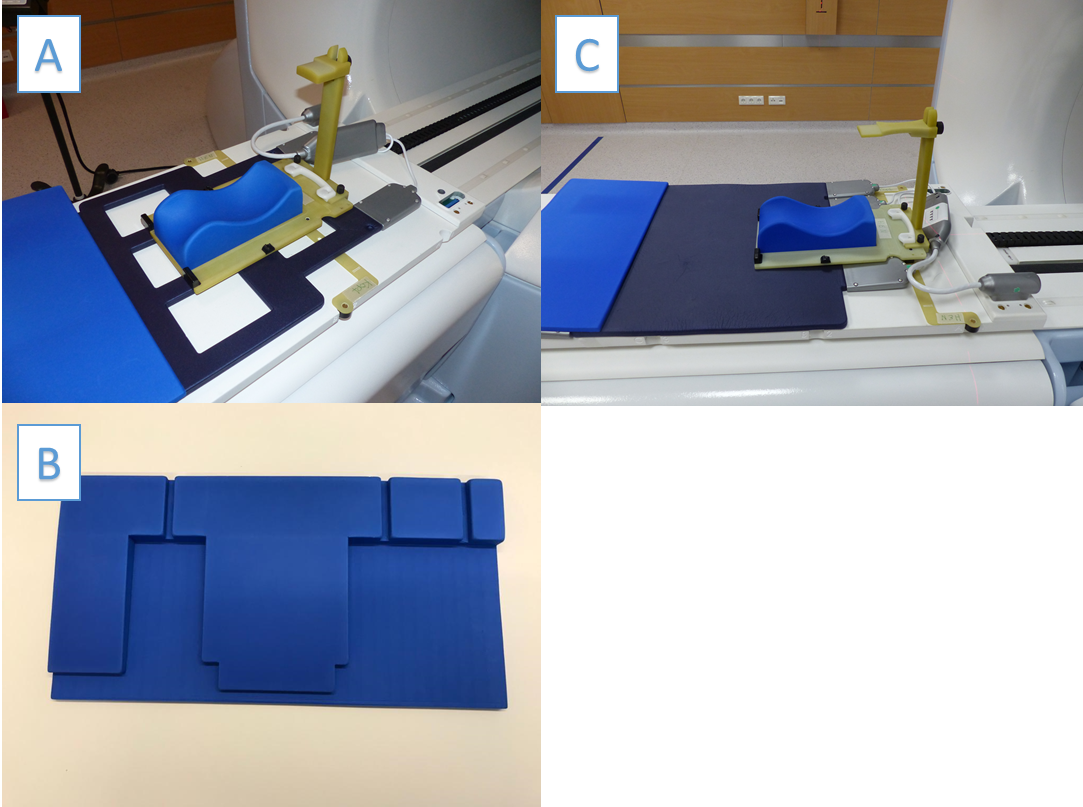
*

*Courtesy of LMU Munich – Viewray MR-Linac. Used devices: HeadSTEP UP VR system for brain (A) or HeadSTEP UP VR H&N system (B) for H&N treatments, HS Flexcoil Holder VR for upper coil positioning, thermoplastic mask, ear plugs, and (C) Flexicoil Comfort Modul VR cushion for flexicoil electronic boxes padding*

1. **Thorax immobilization**

**Table 2 – List of exemplar devices for Thorax immobilization**

| **Therapy region** | **Positioning devices** | **Vendor** | **MR-linac device** |
| --- | --- | --- | --- |
| Thorax | Wing STEP MR system, | IT-V, Innsbruck, Austria | Viewray |
| Thorax | KneeSTEP MR | IT-V, Innsbruck, Austria | Viewray |
| Thorax | Flexicoil Comfort Modul VR cushion for flexicoil electronic boxes padding | IT-V, Innsbruck, Austria | Viewray |
| Thorax | KneeSupport foam, KneeSupport LiftBlock, FluxBoard, Foam mattress MRIdian padding | Macromedics, Waddinxveen, The Netherlands | Viewray |
| Thorax | Headrest M | Elekta, Stockholm, Sweden/ IT-V, Innsbruck, Austria | Unity |
| Thorax | Headrest indexing adapter | Elekta, Stockholm, Sweden/ IT-V, Innsbruck, Austria | Unity |
| Thorax | KneeSupport foam | MacroMedics, Moordrecht, The Netherlands | Unity |
| Thorax | Earplugs E-A-R soft | 3M, USA | Unity |

**Figure 3 – Devices for Thorax immobilization (without patient)**


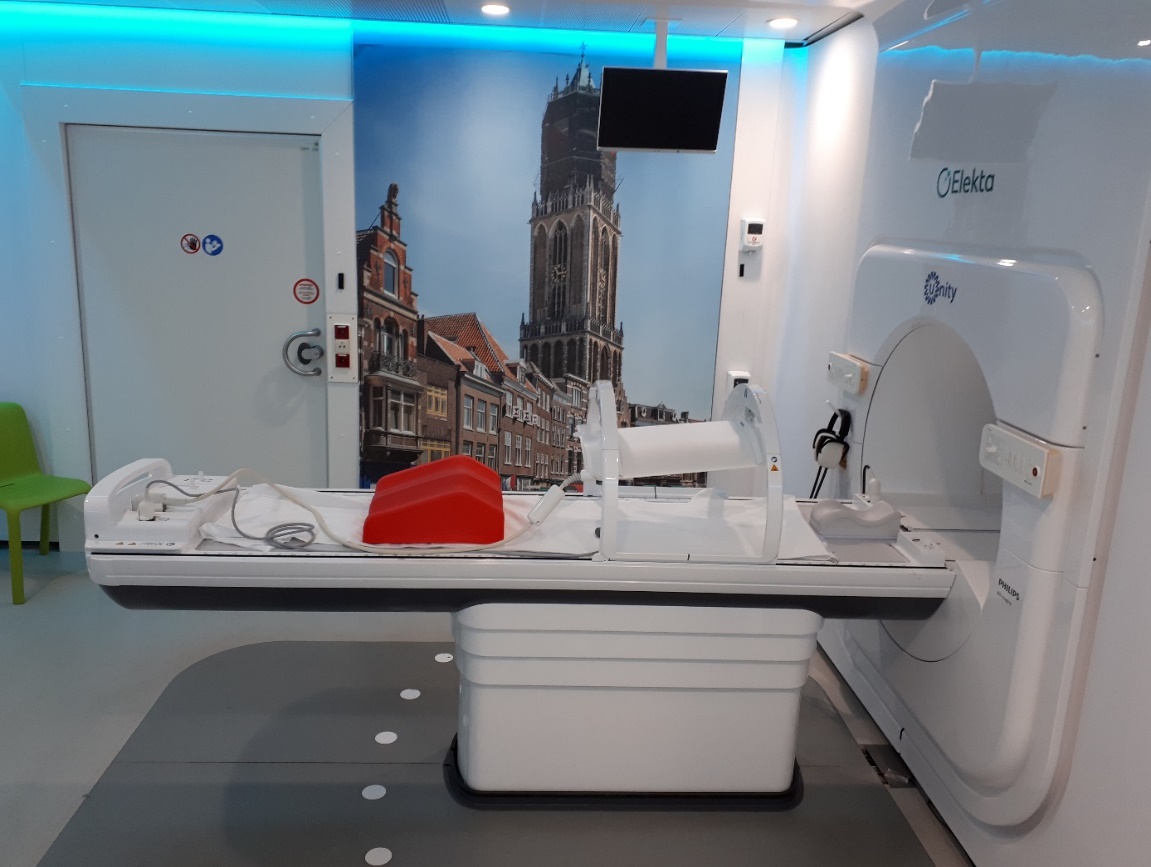


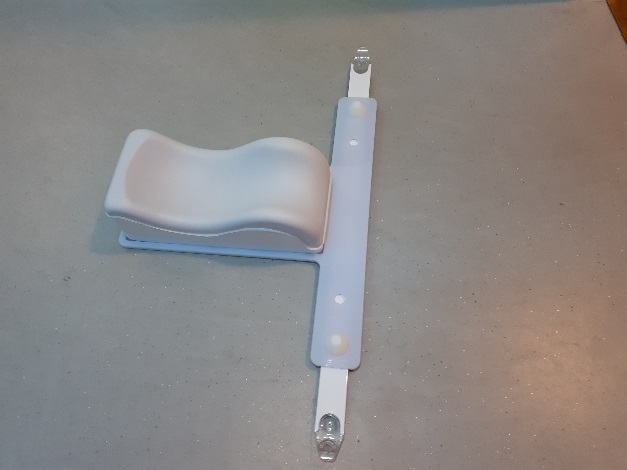


*Courtesy of UMC Utrecht – Unity Elekta MR-Linac. Used devices: Headrest M with Headrest indexing adapter attached to indexingbar, KneeSupport™ foam, anterior Elekta coil bridge, headphones and earplugs, emergency squeeze bulb, optional: prism glasses and BOLD TV screen*

**Figure 4 – Devices for Thorax immobilization (with patient)**


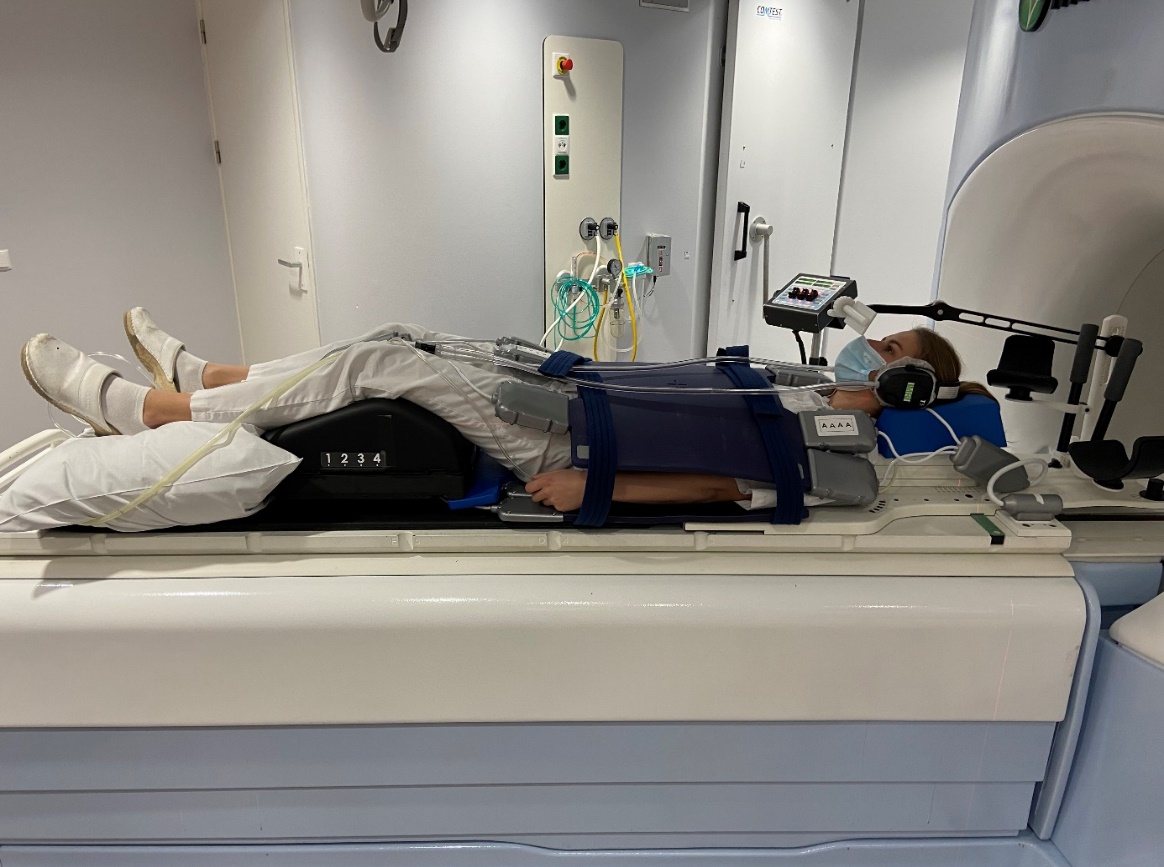


*Courtesy of UMC Amsterdam – MRIdian Viewray MR-Linac. Used devices: KneeSupport foam, KneeSupport LiftBlock, FluxBoard, Foam mattress, MRIdian padding, Hospital pillow, perspex base. Immobilization description: For thoracic and abdominal indications, patients are positioned with both arms parallel to the body. Initial experience with (adaptive) MRgRT with one or both arms above the head indicated that maintaining this position for a prolonged period of time is cumbersome, particularly for elderly patients. To provide visual feedback during breath-hold treatment, a mirror is mounted on the FluxBoard above the patients head. In that manner, patient can observe the monitor which is mounted at the wall at the head end of the MRIdian and actively participate with gated therapy.*

1. **Breast immobilization**

**Table 3 – List of exemplar devices for Breast immobilization**

| **Therapy region** | **Positioning devices** | **Vendor** | **MR-linac device** |
| --- | --- | --- | --- |
| Breast | WingSTEP MR system | IT-V, Innsbruck, Austria | Viewray |
| Breast | KneeSTEP MR | IT-V, Innsbruck, Austria | Viewray |
| Breast | KneeSupport foam, KneeSupport LiftBlock, FluxBoard, Foam mattress MRIdian padding | Macromedics, Waddinxveen, The Netherlands | Viewray |
| Breast | WingSTEP MR system | Elekta, Stockholm, Sweden/ IT-V, Innsbruck, Austria | Unity |

*Courtesy of UMC.*

1. **Abdomen immobilization**

**Table 4 – List of exemplar devices for Abdomen Immobilization**

| **Therapy region** | **Positioning devices** | **Vendor** | **MR-linac device** |
| --- | --- | --- | --- |
| Abdomen | WingSTEP MR system, indexed kneestep | IT-V, Innsbruck, Austria | Viewray |
| Abdomen | KneeSTEP MR | IT-V, Innsbruck, Austria | Viewray |
| Abdomen | KneeSupport foam, KneeSupport LiftBlock, FluxBoard, Foam mattress MRIdian padding | Macromedics, Waddinxveen, The Netherlands | Viewray |
| Abdomen | Fluxboard: U-grup; Head support ; KneeSupport foam; FeetSupport fixed | Macromedics, Moordrecht, The Netherlands | Viewray |
| Abdomen | WingSTEP | Elekta, Stockholm, Sweden / IT-V, Innsbruck, Austria | Unity |
| Abdomen | KneeSTEP | Elekta, Stockholm, Sweden / IT-V, Innsbruck, Austria | Unity |
| Abdomen | ZiFix | QFix, Avondale, USA | Unity |
| Abdomen | BlueBAG BodyFIX 14 Rectangular 700x1825 mm/50L with indexingbars | Elekta, Stockholm, Sweden | Unity |
| Abdomen | Headrest M | IT-V, Innsbruck, Austria | Unity |
| Abdomen | Individual corset | Neofrakt, Spronken Orthopedie NV, Genk, Belgium | Unity |
| Abdomen | KneeSupport foam (fixed in BlueBAG) | MacroMedics, Moordrecht, The Netherlands | Unity |
| Abdomen | Earplugs E-A-R soft | 3M, USA | Unity |

**Figure 5 – Abdomen immobilization**


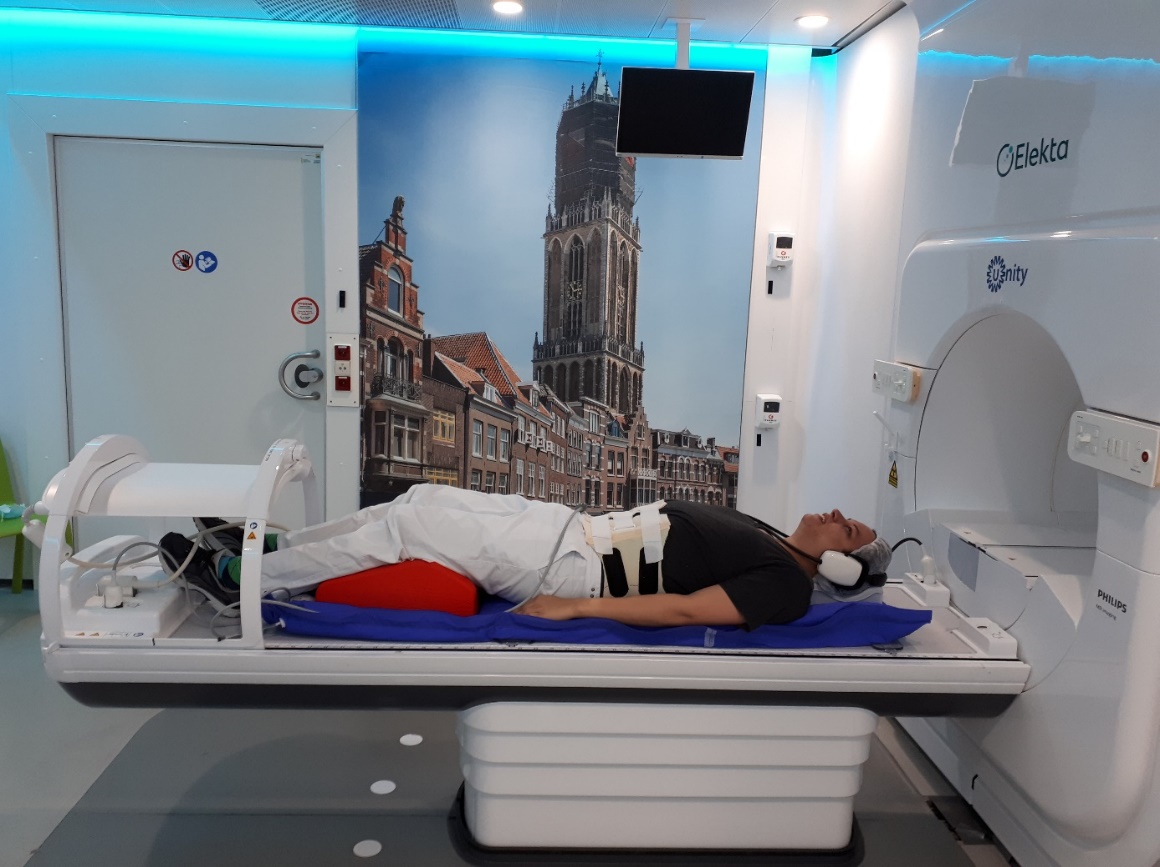


*Courtesy of UMC Utrecht – Unity Elekta MR-Linac. Used devices: BlueBAG™ BodyFIX 14 Rectangular 700x1825 mm/50L, attached with 2 indexingbars on table, Headrest M, fixed in bluebag, KneeSupport™ foam fixed in bluebag, anterior Elekta coil bridge, individual corset, headphones and earplugs, emergency squeeze bulb, optional: Prism glasses and BOLD TV screen*

1. **Pelvis immobilization**

**Table 5 – List of exemplar devices for Pelvis immobilization**

| **Therapy region** | **Positioning devices** | **Vendor** | **MR-linac device** |
| --- | --- | --- | --- |
| Pelvis | ProSTEP PC MR | IT-V, Innsbruck, Austria | Viewray |
| Pelvis | KneeSTEP VR and FeetSTEP VR | IT-V, Innsbruck, Austria | Viewray |
| Pelvis | Flexicoil Comfort Modul VR cushion for flexicoil electronic boxes padding | IT-V, Innsbruck, Austria | Viewray |
| Pelvis | Fluxboard: KneeSupport foam;  FeetSupport fixed | Macromedics, Moordrecht, The Netherlands | Viewray |
| Pelvis | Shoulder Support Cushion | CivcoRT, Iowa, United States | Viewray |
| Pelvis | KneeSTEP and FeetSTEP MR | Elekta, Stockholm, Sweden/ IT-V, Innsbruck, Austria | Unity |
| Pelvis | KneeSupport foam | MacroMedics, Moordrecht, The Netherlands | Unity |
| Pelvis | Plastic Prism glasses with BOLD TV screen | Y&Y Vertical, France.  Cambridge Research Systems, UK | Unity |
| Pelvis | BlueBAG BodyFIX 14 Rectangular 700x1825 mm/50L (with indexingbars) | Elekta, Stockholm, Sweden/ IT-V, Innsbruck, Austria | Unity |
| Pelvis | Headrest M | IT-V, Innsbruck, Austria | Unity |
| Pelvis | Emergency squeeze bulb | Elekta, Stockholm, Sweden | Unity |

**Figure 6 – Pelvis immobilization**


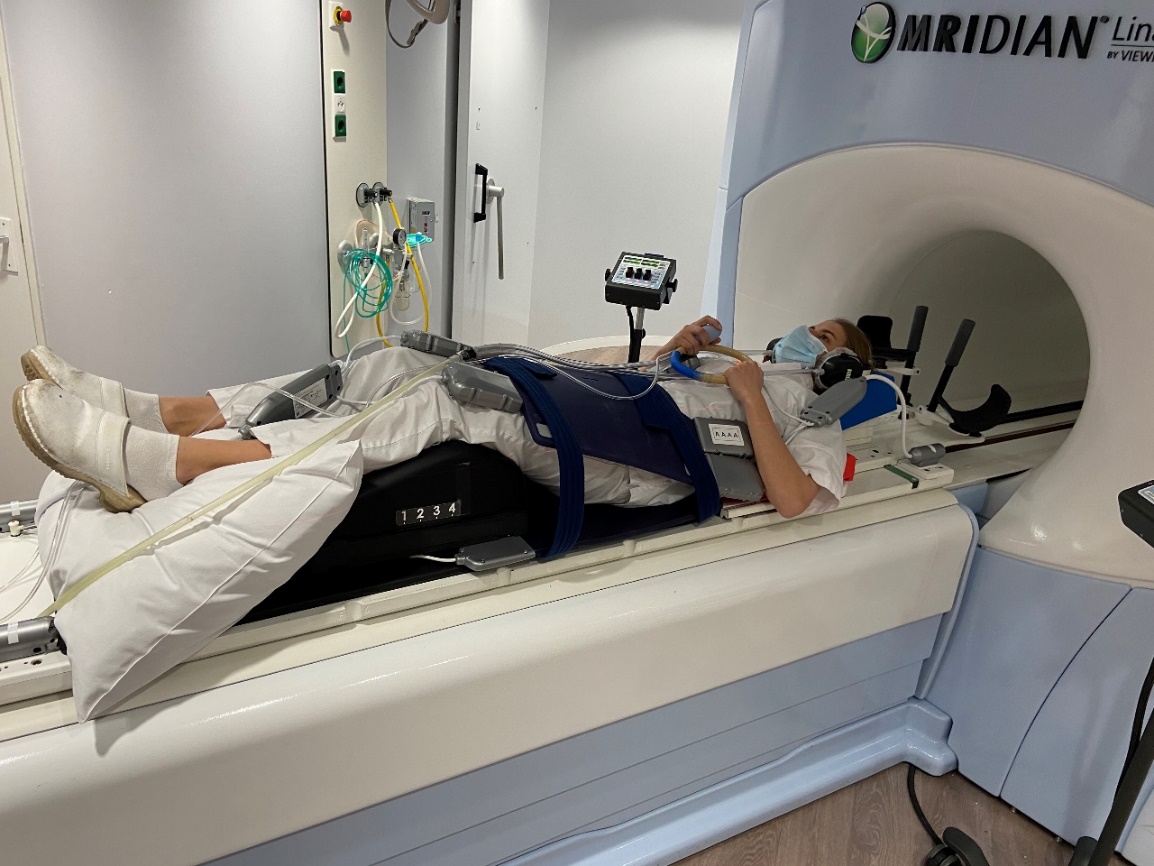


*Courtesy of UMC Amsterdam – MRIdian Viewray MR-Linac. Used devices: KneeSupport foam, KneeSupport LiftBlock, FluxBoard, Foam mattress, shoulder support cushion, Hospital pillow. Immobilization description: Because of the caudal position of the coils for pelvic lesions, the MRIdian padding is not necessary. For comfort, a shoulder support cushion is added to the setup of patients. Additionally, the arms are positioned on the chest with the hands holding a 15 centimeter diameter ring to avoid a closed circuit*

**Figure 7 – Pelvis immobilization**


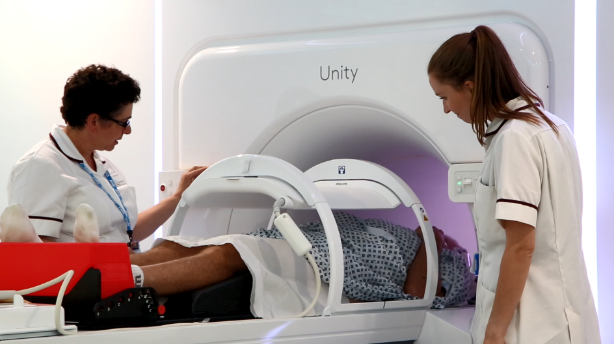


*Courtesy of Royal Marsden NHS Foundation Trust – Unity Elekta MR-Linac;*

**Additional devices for patients positioning: Prism glasses**

Claustophobic patients can be given the option to wear prism glasses during treatment. At the head of the patient, outside the MR bore, is a TV screen (BOLD TV screen, Cambridge Research Systems, UK) on which a video is played. These glasses are not possible for head and neck patients.


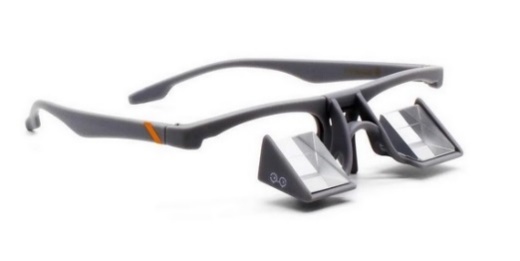

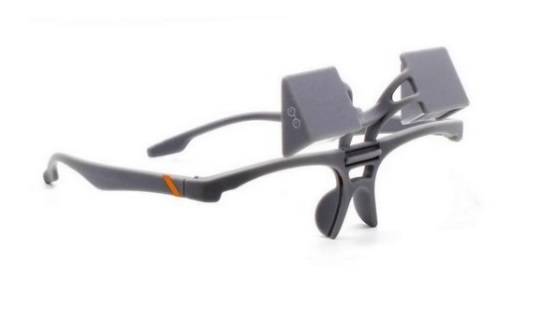

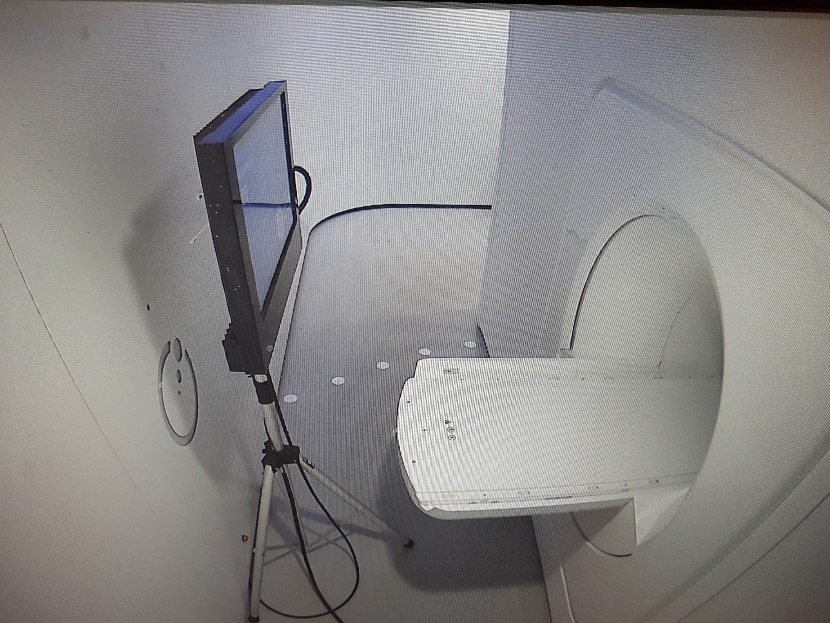


(courtesy of UMC Utrecht)
